# Review of fixation techniques for the four-part fractured proximal humerus in hemiarthroplasty

**DOI:** 10.1186/1749-799X-6-36

**Published:** 2011-07-18

**Authors:** Daniel Baumgartner, Betsy M Nolan, Robert Mathys, Silvio Rene Lorenzetti, Edgar Stüssi

**Affiliations:** 1 Institute for Biomechanics, ETH Zurich, Wolfgang-Pauli Strasse 10, 8093 Zurich, Switzerland; 2 50 N Illinois St 817, Indianapolis, IN 46204, USA; 3 RMS Foundation, Bischmattstrasse 12, 2544 Bettlach, Switzerland; 4 Institute for Biomechanics, ETH Zurich, Wolfgang-Pauli Strasse 10, 8093 Zurich, Switzerland; 5 Institute for Biomechanics, ETH Zurich, Wolfgang Pauli Str. 10, 8093 Zurich, Switzerland

## Abstract

**Introduction:**

The clinical outcome of hemiarthroplasty for proximal humeral fractures is not satisfactory. Secondary fragment dislocation may prevent bone integration; the primary stability by a fixation technique is therefore needed to accomplish tuberosity healing. Present technical comparison of surgical fixation techniques reveals the state-of-the-art approach and highlights promising techniques for enhanced stability.

**Method:**

A classification of available fixation techniques for three- and four part fractures was done. The placement of sutures and cables was described on the basis of anatomical landmarks such as the rotator cuff tendon insertions, the bicipital groove and the surgical neck. Groups with similar properties were categorized.

**Results:**

Materials used for fragment fixation include heavy braided sutures and/or metallic cables, which are passed through drilling holes in the bone fragments. The classification resulted in four distinct groups: A: both tuberosities and shaft are fixed together by one suture, B: single tuberosities are independently connected to the shaft and among each other, C: metallic cables are used in addition to the sutures and D: the fragments are connected by short stitches, close to the fragment borderlines.

**Conclusions:**

A plurality of techniques for the reconstruction of a fractured proximal humerus is found. The categorisation into similar strategies provides a broad overview of present techniques and supports a further development of optimized techniques. Prospective studies are necessary to correlate the technique with the clinical outcome.

## Introduction

### Clinical background

Hemiarthroplasty represents an established treatment method for three or four-part fractured proximal humeri. Pain relief is often achieved by this surgical intervention, but the functional result is less predictable [1,2]. Consequently, clinical outcome scores are ranging from bad-satisfactory to good-excellent (Table 1). Complications such as non-union or resorption of the tuberosity fragments occur in 30-70% of all cases [3-9]. Reasons for this poor outcome may be secondary displacement which negatively affects the muscular balance at the rotator cuff and predisposes the patient to worse clinical results [10-14]. Tuberosity malposition also correlates with fatty infiltration into the rotator cuff and subsequent disuse of the shoulder function [15]. Different patient specific factors such as health status or rehabilitation after surgery influence the result: Injury related variables are predetermined such as the severity of fracture dislocation, neurological deficits or the type of fracture [16]. Although the optimisation of the implant design is often discussed, a significant correlation between a specific prosthesis type and patient satisfaction was not observed [17]. Nevertheless, a significant better Constant Score for one specific fragment fixation technique (using additional cable to the suture fixation) compared to the established technique of using sutures was seen [18]. Other surgeons' experiences support the findings that the fixation technique seems to be crucial for tuberosity union and apparently represents one of the most influencing factors for a good outcome [19-22]. Furthermore, the grade of tuberosity dislocation directly correlates with the clinical outcome. It is therefore assumed that the prevention of fragment dislocation by a stable fixation technique has a direct impact on the clinical result [23].

**Table 1:** Postoperative results of proximal humeral fractures with respect to the Constant Score

Reference	# cases	Follow-up [Mts]	Ø-Age [Years]	Anat. Tuberosity healing	Constant Score
Ambacher et al [32]	27	42	69	**-**	**65**

Becker et al [33]	27	45	67	**-**	**45**

Boileau et al [12]	66	27	66	**-**	**56**

Boileau et al [34]	43	29	68	**15**	**60**

Bosch et al [1]	40	43	68	**-**	**54.2**

Boss et al [17]	20	32	77	**-**	**52**

Christoforakis et al [35]	26	50	65	**-**	**70.4**

Demirhan et al [18]	32	35	58	**26**	**68**

Kollig et al [20]	46	62	60	**-**	**66**

Kralinger et al [13]	167	29	70	**90**	**55.4**

Reuther et al [36]	56	39	71	**28**	**46**

Zyto et al [10]	36	12.4	72	**-**	**57.5**

Loew et al [25]	21	29.3	74.1	**15**	**51.5**

Mehlhorn et al [37]	26	17	70.3	**-**	**52**

Grönhagen et al [19]	46	53	72		**42**

### History of proximal humeral fracture fixation for hemiarthroplasty

The first operation of a shoulder replacement was performed 1893 by Dr. Péan [24]. Horse hairs were used to reattach the muscles to the predrilled holes in the prosthesis shaft. Themistocles Gluck mentioned the fixation of the prosthesis to the bone by different osteosynthesis techniques. However, he did not further analyse the fixation of the fragments in particular [25]. In the modern era, techniques for proximal humeral fragment fixation were established by Neer et al, focussing on the placement of the cables and sutures at the proximal humerus [26]. Current fixation techniques correspond to the appropriate prosthesis designs and are therefore primarily described in detail in the OP manuals of the implant industry.

Published fixation techniques were often tested in a biomechanical test to analyse strength and stability. In prior studies, different in-vitro loading profiles were applied such as load-to-failure testing of fixation techniques [27,28]. In other biomechanical tests, a torque was introduced at the humeral bone which induced a rotation around the humeral longitudinal axis to apply passive muscular tension [29,30]. A further investigation used a numerical approach to mathematically determine the strength of the fixation by means of a Finite Element Analysis [31]. These biomechanical investigations show that substantial efforts have been made to find an appropriate and stable fixation technique for a four-part fracture. Nevertheless, a comprehensive collection of existing techniques is needed prior to biomechanical testing.

A summary of existing fixation techniques may support the identification of further advantageous techniques. By comparing the most frequent techniques, promising features and innovative procedures may be combined. Existing publications focus primarily on one specific technique; it is therefore of interest to have a direct comparison. Classifying the different techniques in distinct groups supports a schematic innovation process to develop novel techniques. The aim of this investigation is therefore the analysis of existing fixation techniques for proximal humeral four-part fractures for hemiarthroplasty.

### Method of analyzing fixation techniques

**Figure 1:**
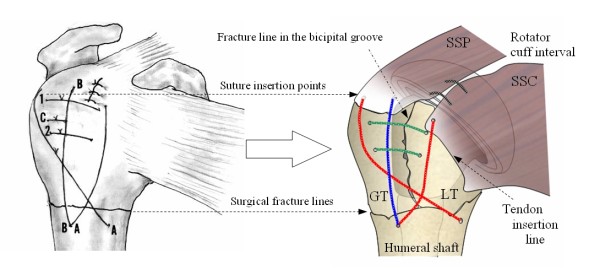
**Transfer of one published fixation technique (left, Dines et al) into a schematic representation (right) by using described anatomical landmarks**. (Reproduced with permission of the author, Images copyright 2002, Joshua S. Dines. MD).

A review of the different fixation techniques in the literature was carried out focusing on proximal humeral four-part fractures. Suture and wire placement based on illustrations from literature (Figure 1, left) was transferred in a standardised image demonstrating a restored rotator cuff in anteriolateral view (Figure 1, right). Anatomical landmarks at the proximal humerus such as the bicipital groove, the surgical neck fracture line, tendon insertions and the rotator cuff interval were used to localise the suture configurations and the placement on the bone surface. For simplicity, all left shoulders have been inverted to standardize all techniques to the right shoulder. In our opinion, this procedure represents a reliable method, as the mentioned anteriolateral view is frequently used to represent performed fixation techniques. The data recorded include:

- the number of strands connecting the humeral shaft to the greater tuberosity;

- the number of strands connecting the humeral shaft to the lesser tuberosity;

- the number of strands connecting the greater and lesser tuberosity to each other;

- the number of strands connecting the greater and lesser tuberosity to the shaft;

- the design of the middle parts of corresponding prosthesis including

- the number of holes and fins in the proximal shaft

- the qualitative prosthesis shape

### Conventions

A uniform terminology of suture placement was defined that corresponds to the frequently used conventions in literature: the strands oriented parallel/collinear to the shaft axis were defined as longitudinal, leading from proximal-to-distal. Circular strands were perpendicular to the longitudinal axis of the humerus, placed circumferentially around the cuff. Transverse sutures represented a placement through the prosthesis. Diagonal sutures were guided from the GT-LT fragment to the anterior-posterior diaphysis of the shaft. The use of dotted lines represented transosseous sutures. Blue lines represented a tuberosity connection to the shaft, and green lines represented interfragmentary connections between the LT and the GT. Cerclages around the GT and LT, guided through the prosthesis, are shown as magenta, and metallic braided cables as black. Sutures interconnecting all three fragments like GT, LT and shaft are shown as red.

### Review

Several investigations have applied the figure-of-eight technique by interconnecting all three fragments such as the shaft, the GT and the LT [32-34]. Dines et al recommended the attachment of the tuberosities to the shaft, to each other, and to the fin of the prosthesis (Table 2). First the GT is secured to the shaft and to the fin of the prosthesis using transverse sutures. Then the LT is fixed to the shaft and to the GT. With the tuberosities now secured to the prosthesis stem, a figure-of-eight tension band is placed through the rotator cuff tendons near their insertion into the tuberosities, and finally tied to the proximal shaft. A longitudinal suture is used for an additional fixation of the GT to the shaft. The posterior longitudinal suture enters in the superior portion of the supraspinatus tendon and is connected to the shaft. Hence, the GT is secured to the shaft with a separate suture.

**Table 2:** Schematic overview of performed fixation techniques and corresponding implant designs

Reference	Graphics	Prosthesis			Fixation Technique			
			**# fins**	**# holes**	**# of strands GT-shaft**	**# of strands LT-shaft**	**# of strands LT-GT**	**# of strands LT-GT Shaft**

**(Frankle and Mighell 2004)**			2 fins	No holes	1	1	2	0
					
					**Neer III **, Smith&Nephew Two internally placed augmentation sutures Vertical cross-stitches			

**Dines 2002** **Abrutyn 2003**		-		-	2	1	2	0
					
					No remarks of implant type			

**Boileau OP-Manual**			No fiSns	One central hole	0	0	2	2
					
					**Aequalis**, Tornier			

**Voigt 2007**			2 fins	4 holes each	1	1	3	0
					
					Two figures-of-eight tuberosities fixed at the head support. **Univers**, Arthrex			

**Gerber OP-Manual**			No fins	2 holes	1	1	2	0
					
					**Anatomical Fracture**, Zimmer			

**Krause 2007 Hertel**			No fins	2 holes	0	0	1	2
					
					Cable system for the entire fixation **Epoca**, Synthes			

**Reuther 2008**			No fins	2 holes	1	1	2	0
					
					Cable system around the GT-LT prosthesis **Affinis Fracture**, Mathys Medical			

**Beutler De Wilde, Poster**			No fins	3 holes	1	1	2	1
					
					**Epoca**, Synthes			

Similar to the previous technique of Dines et al, the technique of Frankle et al uses the same prosthesis type [35,22]. Both tuberosity fragments are fixed to the middle part of the prosthesis. A circumferentially oriented suture secures the tuberosities to each other: one end of the suture captures the GT by placing it through the posterior rotator cuff, whereas the opposite end captures the LT. The circumferential suture is first tied to fix the tuberosities together. Drill holes are placed distally to the surgical neck for reattachment of the tuberosities to the shaft in a figure-of-eight technique. These longitudinal sutures are then finally tied to secure the tuberosities to the shaft. The Aequalis fracture prosthesis is used in another current technique by Boileau et al [19]. Two sutures are placed in the ISP and two in the Teres Minor (TM) tendon. Reconstruction starts with the first two of a total of four circular sutures. These are passed around the prosthetic neck to fix the GT. Then the LT is fixed by using the other two circular sutures. The two lower sutures are subsequently fixed to the tendon insertion to pull the rotator cuff distally and restore the pretension on the rotator cuff tendons. Translational and rotational tests have been performed to assess the stability of fixation. Large-diameter (no. 5 or 7) non-absorbable sutures were used to secure both tuberosities. Circular and longitudinal sutures secure the fragments with respect to a potential multidirectional muscle tension.

In contrast to the previously discussed fixation techniques, both tuberosities may be fixed individually to the shaft by separate figure-of-eight tension bands [36]. The Univers prosthesis is used in Voigt's description which has lateral fins. Two holes are drilled in the posterior and anterior humeral shaft to reduce each of the tuberosities. Three circular sutures are initially positioned around the greater tuberosity and the prosthetic neck. The lesser tuberosity is held by two sutures passed through the anterior-medial holes of the prosthesis. The circular sutures are first tied to pull down both tuberosities into the anatomical position. A technique similar to that of Voigt et al has been performed by Gerber et al. [37]. In this technique using the Anatomical fracture prosthesis (Zimmer Ltd), sutures are placed in both tuberosities to pull them down to the shaft. This prosthesis design does not provide fins, which affords more room proximally for tuberosity positioning.

First, the circular sutures connecting the tuberosity fragments are tied, then the strands to the shaft are tightened. A suture is placed in a predrilled GT hole and a second one in the LT hole. A cerclage suture is passed through the SSC tendon, around the GT and the LT and ends at the ISP and TM tendon insertion. A suture in the humeral shaft, medial to the bicipital groove, pulls the distal end of the lesser tuberosity back down to the shaft. Additional sutures in the middle of the prosthesis are used for further reduction.

Reuther et al. use the Affinis fracture prosthesis (Mathys Ltd) [38]. To achieve a better tuberosity fixation, the central part of the prosthesis is equipped with two holes to insert non-absorbable sutures or cables. The central part does not have any fins and is covered by rough calcium phosphate coating. After pulling through the sutures, the tuberosities are height-adjusted and fixed with retention stitches to the outer edge of the central part and over each other. Both tuberosities are fixed to the stem by circumferential wiring. Finally, the circular compression cable (grey) is closed.

In the technique of Hertel et al, fixation consists only of metallic cables, without using sutures [39]. This method is applied together with the Epoca prosthetic system (Synthes GmbH, Switzerland), which has a rectangular shaft design including two anteroposterior holes, but no fins. Two horizontal wires connect the fragments to the prosthesis. The titanium cables are pulled by a tensioner and closed by a clamp mechanism.

A tension-band technique using braided polyester sutures has been used for biomechanical testing [28] using the Epoca prosthesis. The tuberosities are fixed to the rim of the prosthetic head via sutures passed through the tendon-to-bone junction. In addition, the tuberosities are sutured to each other and to the humeral diaphysis. Circular, transosseous sutures connect both tuberosities. The tuberosities are fixed to the diaphysis with longitudinal single-loop sutures.

Similar fixation technique has also been applied to the humerus reconstruction without using an implant [40]. Such configurations are also applicable for hemiarthroplasty and were therefore considered too. The study shows a treatment for a four-part, valgus impacted fracture. Tuberosities are secured to each other and to the medial and lateral side of the diaphysis in a cruciate fashion. Another two pairs of sutures are inserted laterally and medially through drill holes in the diaphysis. These sutures are guided into the opposite tuberosity, near the musculotendinous junction. Each suture is tied individually and then to each other in a cruciate arrangement.

Stability was investigated in a biomechanical test for three different fixation techniques by Abu Rajab et al [27]. The monobloc Neer prosthesis design with two lateral fins and four suture-wire holes was used. In the first technique, both tuberosities were attached to the shaft and to each other, each with separate sutures. In the second technique, an additional cerclage is placed through the medial fin. Biomechanical testing revealed that an additional cerclage does not enhance the stability but that the stability was significantly reduced if the tuberosities were not fixed to each other.

Metallic wires are also used for a figure-of-eight tension band technique [41]. The anterior wire fixes the lesser tuberosity and the attached subscapularis muscle, the superior one passes through the supraspinatus tendon and around the greater tuberosity back to the shaft. Whereas Wijgman et al placed the cerclage wires as close to the tendon insertions as possible, others prefer a transosseous placement of the cable through the tuberosities [42-44]. This difference results from two philosophies: wires may have a negative influence on the periosteal blood supply, particularly in a vascular area such as the rotator cuff.

Recent hemiarthroplasty treatments are using a modified prosthesis' middle part. Schittko et al propose a middle part with multiple holes for an unconstrained placement of the tuberosities using the Ortra prosthesis [45]. A further method of tuberosity reconstruction is presented by Sosna et al [46], where humeral plating and hemiarthroplasty is combined. A screw inserted into a proximal plate (fixed to the prosthesis), through the tuberosities into the prosthesis middle shaft provides primary fragment stability. A summary of all described techniques is given in Table 2.

## Results

Based on the analysis in the review, four groups of different fixation techniques are built. Each group summarises therefore a similar strategy of fixation:

Group A: Tuberosities and shaft are connected by one single suture. In group A, Dines et al and Frankle et al use a figure-of-eight tension band over the entire surface of the rotator cuff to connect all three fragments such as the humeral shaft, the GT and the LT.

Group B: The single tuberosities are independently connected to the shaft and among each other: In group B, Voigt et al and Gerber et al use the figure-of-eight tension band to connect only single fragments independently to the stem, without involving all three fragments. Voigt et al, Boileau et al and Reuther et al place several sutures ranging from the SSC to the ISP tendon insertion and additionally apply a tension-band technique between the LT and the GT in a horizontal orientation.

Group C: Metallic cables are used in addition to the sutures: Reuther et al, De Wilde et al and Hertel et al use metallic cables either applied alone or in combination with sutures. The cables are often placed circumferentially around the shaft humeral shaft.

Group D: Short suture loops are used to connect adjacent fragments together. The suture loops are placed close to their fragment borderlines.

## Discussion & Conclusion

Recently, the fixation technique of proximal humeral four-part fractures is often discussed in literature. The number of acquired references demonstrates the high relevance in fixing humeral proximal four-part fractures.

Generally, securing tuberosity fragments against medial displacement is done by horizontal sutures, circumferentially around the cuff. The sutures are passed around the prosthesis and the humeral long bone in form of a closed-loop. A similar placement is done for the metallic cables. No anchor in the bone is therefore needed, since two suture/cable ends are fixed together by knots.

Placement of stable sutures along the humeral axis on the bone surface - connecting the shaft to single tuberosities - seems to be more challenging: Drilling holes are needed in the shaft and in the fragments as anchor points. The effect of cutting-out of sutures through the bone has to be expected. Due to the fact that proximal fragment displacement is seen clinically, the scenario of a cutting effect has to be assumed. Further studies discuss a fixation of sutures at the proximal humeral shaft without using drilling holes [47]

**Figure 2:**
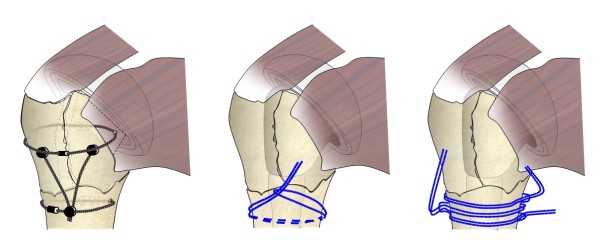
**Anchoring cables by bone washers according to Baumgartner et al (left) or placement of sutures circumferentially around the proximal humeral shaft, Pijls et al (middle and right)**.

To be able to strengthen proximal-to-distal tuberosity-to-shaft connections, a placement of a cable along the humeral axis would be of interest. Connecting two circumferential oriented cables (one placed around the fragments, one around the shaft) by another cable presumably leads to enhanced stiffness [48] (Figure 2).

Further prospective studies are necessary to correlate specific techniques with the clinical outcome. A standardised, biomechanical testing strategy according to physiological loads is needed to evaluate the strength of such techniques.

## List of abbreviations

LT: Lesser Tuberosity; GT: Greater Tuberosity; SSP: Supraspinatus; ISP: Infraspinatus; SSC: Subscapularis; TM: Teres Minor.

## Competing interests

The authors declare that they have no competing interests.

No fees or funding was received from a commercial partner.

## Authors' contributions

DB as the main author was responsible for the preparation of the manuscript.

The technical analysis of existing fixation techniques was provided by BN, RM and SL. ES is the head of the department and approved the strategic background of present publication. All authors read and approved the final manuscript.
